# Comparison of the Simple Cyclic Voltammetry (CV) and DPPH Assays for the Determination of Antioxidant Capacity of Active Principles 

**DOI:** 10.3390/molecules17055126

**Published:** 2012-05-03

**Authors:** Jesús F. Arteaga, Mercedes Ruiz-Montoya, Alberto Palma, Gema Alonso-Garrido, Sara Pintado, José M. Rodríguez-Mellado

**Affiliations:** 1 Department of Chemical Engineering, Physical Chemistry and Organic Chemistry, University of Huelva, Campus de El Carmen, 21071 Huelva, Spain; 2 Department of Physical Chemistry and Applied Thermodynamic, Córdoba University, Campus de Excelencia Internacional Agroalimentario (CEIA3), ed. Marie Curie, 14014 Córdoba, Spain

**Keywords:** antioxidant activity, cyclic voltammetry, DPPH, phenolics, low weight antioxidants

## Abstract

Antioxidant activity of a number of small (low molecular weight) natural compounds found in spices, condiments or drugs (gallic acid, sesamol, eugenol, thymol, carvacrol, vanillin, salicylaldehyde, limonene, geraniol, 4-hexylresorcinol, *etc.*) has been evaluated using electrochemical and DPPH^•^ radical scavenging measurements. Structural analysis of the tested compound suggest a remarkable activity for phenol derivatives and the importance of the –R groups located on the phenolic ring in the molecule’s ability to act as free radical scavenging as well as their influence in the electrochemical behavior. The voltammetric method can be used for the determination of the antioxidant capability in the same manner as the DPPH^•^ radical scavenging because of the correlation found between oxidation potentials and anti-radical power (ARP = 1/EC_50_). Such electrochemical determination is fast and cheap and allows making measurements under a variety of experimental conditions. The accuracy of the electrochemical measurements is the same for all the compounds, irrespective of their scavenging activity, the opposite of what occurs in the DPPH^•^ test.

## 1. Introduction

The search for and use of natural and dietary antioxidants is growing because the public’s perception of their safety *versus* synthetic analogues [1,2]. Since ancient times, spices and condiments have been considered indispensable in the culinary arts, and, in addition, they have been recognized for their physiological and medicinal properties, and their broad-spectrum of effectiveness [3,4]. The antioxidant capability of these compounds, specially phenols [5], towards free radicals normally produced by cell metabolism or in response to external factors is due to the scavenging of free radicals and reactive oxygen species (ROS), which are made inactive [6], thus avoiding or preventing degenerative disorders caused in humans by oxidations of nucleic acids, proteins or lipids [7,8]. Plant phenolics arguably deserve a special mention when one considers that the wide-ranging benefits they offer to plants and hence to other living organisms are essentially all a result of their inherent physicochemical properties bundled within the phenol functional group.

Different assays have been used to evaluate the antioxidant activity of natural products [9,10], but a comparison of the results is very difficult because of the different experimental methods adopted. Antioxidant activities of pure compounds and plant extracts have been determined, among others, by an accelerated test [11,12], by using radical species such as ABTS^+^^•^ [13] and DPPH^•^ [14], by the ESR spin trapping technique and by measuring the oxygen consumption in a heterogeneous lipid/water emulsion with lipid oxidation initiated by metmyoglobin [15]. However, all these procedures present some drawbacks since they require the use of specific reagents and tedious and time consuming sample preparation.

Electrochemical measurements have advantages for the determination of antioxidant activity [16] such as their use as a rapid proof of the antioxidant capacity of a lot of organics. The oxidation potentials measured by cyclic voltammetry (CV) have been used to compare the antioxidant strength of compounds such as phenolic acids, flavonoids, cinnamic acids, *etc.* [16,17,18,19,20], being the glassy carbon electrode (GCE) the more frequently used electrode. Low oxidation potentials are associated with a greater facility or strength of a given molecule for the electrodonation and, thus, to act as antioxidant. There are some papers in the literature testing antioxidant capacity by using electrochemical measurements. Cyclic voltammetry at the GCE has been successfully applied to analyze antioxidants present in wine [21], plant extracts [22], phenolic standards [16] and even human plasma [20]. In these studies the most used parameter was the oxidation potential on the GCE, but this parameter strongly depends on the mechanism of the electrode reaction. The main drawback of the CV assay is that it is properly used to effectively characterize the reducing ability and reversibility of compounds either pure or presents in a real matrix. This value cannot always be directly related with the antioxidant ability of the sample. Besides, in the case of natural samples, the presence of compounds such as sugars or natural polymers could hinder the experimental manipulation as well as interfere with the interpretation of the potential values obtained. There are other studies comparing the data measured by cyclic voltammetry with those obtained by other methods [17,18,19,20,23,24,25,20,23]. The correlations are not always good [17], especially when the voltammetric and DPPH^•^ assays were compared [24]. This last method evaluates the antioxidant activity of a given compound or a complex matrix by reaction in methanolic solution with a stable radical, namely 2,2-diphenyl-1-picrylhydrazyl (DPPH^•^), which has an unpaired valence electron at one atom of its nitrogen bridge [25], the decrease of the DPPH^•^ concentration is measured from the decrease of absorbance at a characteristic wavelength. The correlation between the structure of the low molecular weight antioxidants, especially phenolics, and its antioxidant activity is not well determined at present, being the subject of intense research.

In this paper, the electrochemical behaviour and the antioxidant effectiveness of a number of bioactive compounds are evaluated comparatively by means of the DPPH^•^ test and cyclic voltammetry. It is intended to establish a relation between antioxidant capacity and oxidation potential to substitute the time-consuming DPPH^•^ test by a rapid voltammetric determination. The accumulation of data of this kind is expected to be useful for an improved understanding of the role and activity of organic molecules as antioxidants, and the article would benefit from the analysis of a much larger number of organic molecules of these types.

## 2. Results and Discussion

**Figure 1:**
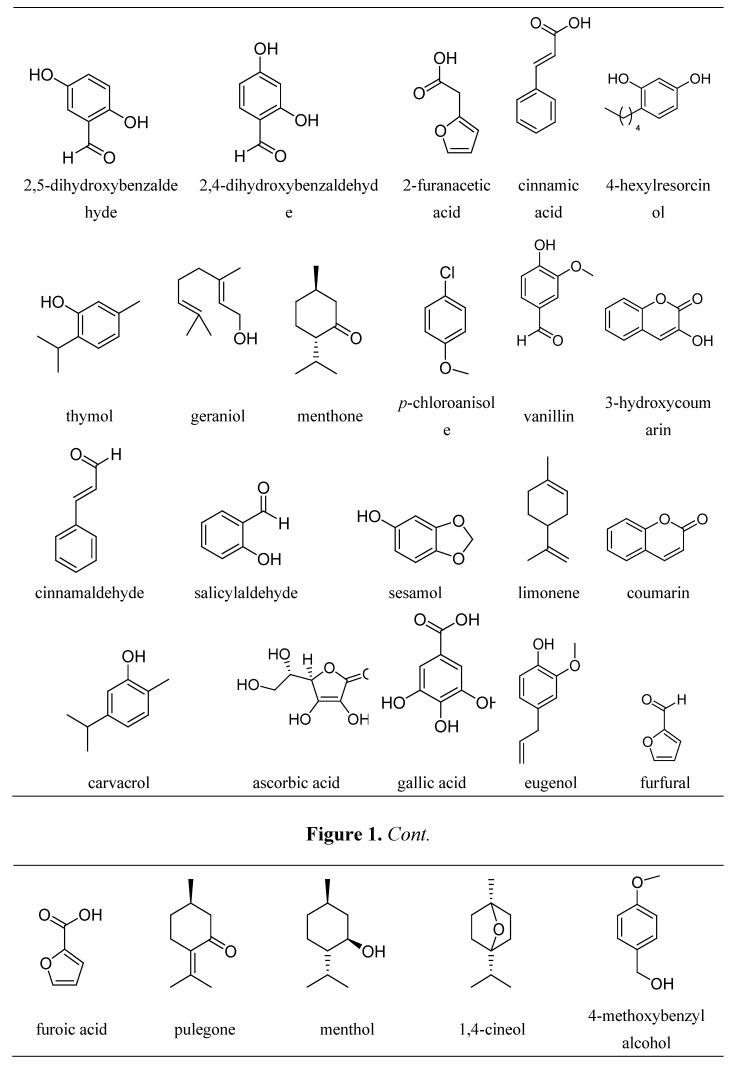
Structures of most of the antioxidants studied.

The compounds studied, whose structures are given in Figure 1, are mainly active principles of spices, seasonings or drugs, belonging to the family of low molecular weight antioxidants that are aromatic phenolics and non-phenolics, or cyclic and acyclic non-aromatic compounds, whose activities are based on their reducing properties.

The main experimental problem of this method arises from the determination of the steady state concentration, since even for antioxidant species having a fast kinetics, after 24 h the decrease of absorbance continues, though at a low rate, and so it is difficult to obtain reproducible measurements. It must be noted that low uncertainties in EC_50_ can cause important changes in the ARP values; the inaccuracies derived from these measurements may provoke alterations in the order of antioxidant activity for a given family of compounds. It was considered that the steady state was reached when the absorbance remained constant during at least 10 min in the uncertainty limits of the spectrophotometer, *i.e.* ±0.001 absorbance units. The results obtained are gathered in Table 1. 

**Table 1:** Values of ARP and electrochemical parameters of the voltammograms recorded for the compounds studied.

Compound	ARP ^[a]^	E_p,a_^[b]^/mV	Compound	ARP ^[a]^	E_p,a_^[b]^/mV
gallic acid (GA)	12.5	274	2,4-dihydroxybenzaldehyde (2,4-BZ)	<10^−3^	841
sesamol (SE)	5.5	343	2,5-dihydroxybenzaldehyde (2,5-BZ)	17.5	202
eugenol (EU)	5.0	411	furfural (FU)	<10^−3^	― ^[c]^
4-hexylresorcinol (HR)	2.3	453	vanillin (VA)	0.11	571
thymol (TH)	0.78	529	cinnamic acid (CA)	<10^−3^	552
carvacrol (CC)	0.12	552	4-methoxybenzyl alcohol (4-MA)	<10^−3^	― ^[c]^
cinnamaldehyde (CI)	<10^−3^	588	ascorbic acid (AA)	6.39	79
3-hydroxycoumarin (HC)	<10^−3^	763	3,5-dimethoxybenzaldehyde (3,5-BZ)	<10^−3^	― ^[c]^
salicylaldehyde (SA)	<10^−3^	860	furoic acid(FA)	<10^−3^	― ^[c]^
coumarin (CU)	<10^−3^	― ^[c]^	pulegone (PU)	<10^−3^	― ^[c]^
geraniol (GE)	<10^−3^	― ^[c]^	menthol (MT)	<10^−3^	― ^[c]^
limonene (LI)	<10^−3^	― ^[c]^	1,4-cineole (CN)	<10^−3^	― ^[c]^
*p*-chloroanisole (CL)	<10^−3^	― ^[c]^	2-furanacetic acid (FN)	<10^−3^	― ^[c]^
benzaldehyde (BZ)	<10^−3^	― ^[c]^	menthone (MT)	<10^−3^	― ^[c]^

^[a]^ ±0.5% = average of ARP confidence interval ^[b]^ E_p,a_, oxidation peak potential; experimental conditions: 5 × 10^−4^ M, pH = 7.00; c v = 0.1 V/s; confidence interval for E_p,a_ values was always lower than ±4 mV; ^[c]^ These compounds showed no peaks in cyclic voltammetry and did not cause any effect on the spectra of DPPH^•^ radical.

These results were not exactly the same as those found in the literature for some of these compounds [26] although they retain the same order. These differences may be explained by the difficulty of obtaining reproducible measurements as just discussed. In the case of CV, small discrepancies on E_p,a_ values [17,19] may be simply due to minimal changes in the experimental conditions (concentration, pH, scan rate, temperature) in which the voltammograms have been registered.

Cyclic voltammograms were recorded for all the compounds studied at different pH values and varying the scan rate and the substrate concentration. An initial assessment of the results allowed us to note that for non-phenolic compounds any signal was observed in CV, while DPPH^•^ value is too small to be determined (Table 1). This allowed us to discriminate those molecules that should not act as prooxidants (which suffer the electrochemical phenomenon), while did not let us to know which of them, even being poorly active, can act as radical scavengers, as the inaccuracy in the measurement of ARP is large enough to determine values of molecules with low level of activity.

**Figure 2:**
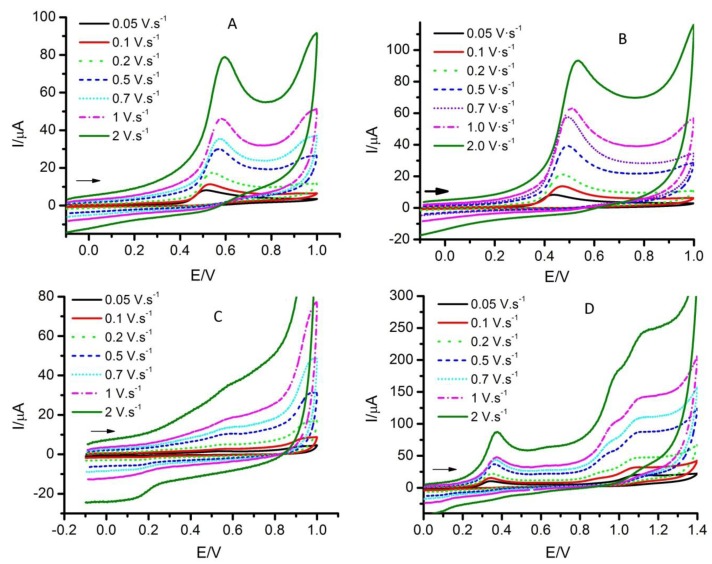
Cyclic voltammograms of A: thymol, B: 4-hexylresorcinol, C: cinnamaldehyde, D: sesamol. Experimental conditions: pH = 7.0, c = 5 × 10^−4^ M and different scan rates. Arrows indicate the initial direction of the scan.

Figure 2 shows some examples for selected substrates at constant reactant concentration and pH. As can be seen, the intensities of the voltammograms increase as the scan rate was increased. So, a higher scan rate implies a higher sensibility, but the charging current also increases. The peak potentials of the oxidation signals shifted in all cases towards positive values, as can be seen in Table 2. The lower dependences were found for the oxidation peaks of HC, 2,4-BZ and SA, in the order of 15 ± 1 mV, followed by 2,5-BZ, SE, CI and EU, in the order of 25 ± 2 mV, being higher for the rest.

Moreover, at high scan rates the voltammograms presented distorted shapes. This could be related with the dependence of the oxidation peak current with the logarithm of the scan rate, which must be close to 0.5 for a diffusion-controlled process. This is accomplished approximately only for SE, TH and HC (Table 2), but in most cases the experimental value is greater than 0.5. These facts can be explained if adsorption processes are involved in the oxidations, causing the distortion at high scan rates and the dependences of the peak potentials towards positive values. This must also be reflected in the dependence of the voltammograms with the reactant concentration.

**Table 2:** Electrochemical parameters of the voltammograms recorded for the active compounds studied.

Parameter [*]	2,5-	GA	SE	EU	HR	TH	CC	VA	CI	HC	2,4-	SA
	BZ										BZ	
E_p,a_/mV	202	274	343	411	453	529	552	571	588	763	841	860
E_p,c_/mV ^[a]^	−11	―	―	71	―	―	―	―	―	―	―	―
ΔE/mV ^[a]^	213	―	―	340	―	―	―	―	―	―	―	―
I_p,a_/µA ^[a]^	35.55	16.23	15.37	11.94	15.34	11.34	7.803	15.52	3.083	10.86	25.52	13.76
I_p,c_/µA ^[a]^	11.25	―	―	2.18	―	―	―	--	―	―	―	―
E_p/2_/mV ^[a]^	163.2	167.4	290.9	361.4	389.6	477.1	473.8	473.2	342.5	756	709.9	643.5
E_p,a_-E_p/2_/mV ^[a]^	38.8	106.6	52.1	49.6	63.4	51.9	78.2	97.8	245.5	104	131.1	92.5
∂log I_p,a_/logv ^[b]^	0.645	0.351	0.535	0.728	0.725	0.575	0.722	0.678	0.753	0.594	0.630	0.622
∂E_p,a_/logv ^[b]^	21.15	146.89	24.33	27.70	56.69	50.73	40.89	9.42	24.35	14.63	14.00	16.27
∂E_p,a_/logc ^[c]^	−1.12	130.13	3.06	20.00	−42.48	28.68	−55.38	29.57	13.16	11.5	7.44	−49.25

^[a]^ 5 × 10^−4^ M, pH = 7.00; c v = 0.1 V/s; ^[b]^ 5 × 10^−4^ M, pH = 7.00; ^[c]^ pH = 7.00; c v = 0.1 V/s. [*] Acronyms of Table 2: E_p,a_, oxidation peak potential; E_p,c_, reduction peak potential; ΔE = E_p,a_ − E_p,c_; I_p,a_, anodic peak current; I_p,c_, cathodic peak current; E_p/2_, potential to a current value corresponding to half of I_p,a_.

**Figure 3:**
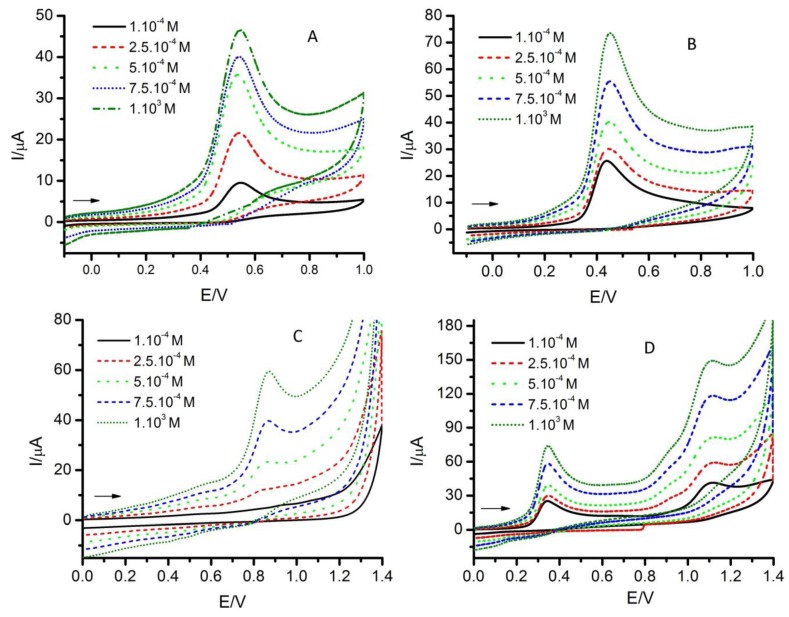
Cyclic voltammograms of A: carvacrol, B: 4-hexylresorcinol, C: salicylaldehyde, D: sesamol. Experimental conditions: pH = 7.0, v = 0.1 V·s^−1^ and different concentrations. Arrows indicate the initial direction of the scan.

Figure 3 shows some examples for selected substrates at a constant scan rate and pH and, as can be seen, the intensities of the voltammograms increase as the antioxidant concentration increases. In this case, a higher concentration implies a higher sensibility, but the charging current increases also, as occurred when the scan rate was increased. The peak potentials of the oxidation signals shifted in all cases towards positive or negative values, as can be seen in Table 2, with the exception of SE, for which the variation can be considered null.

**Figure 4:**
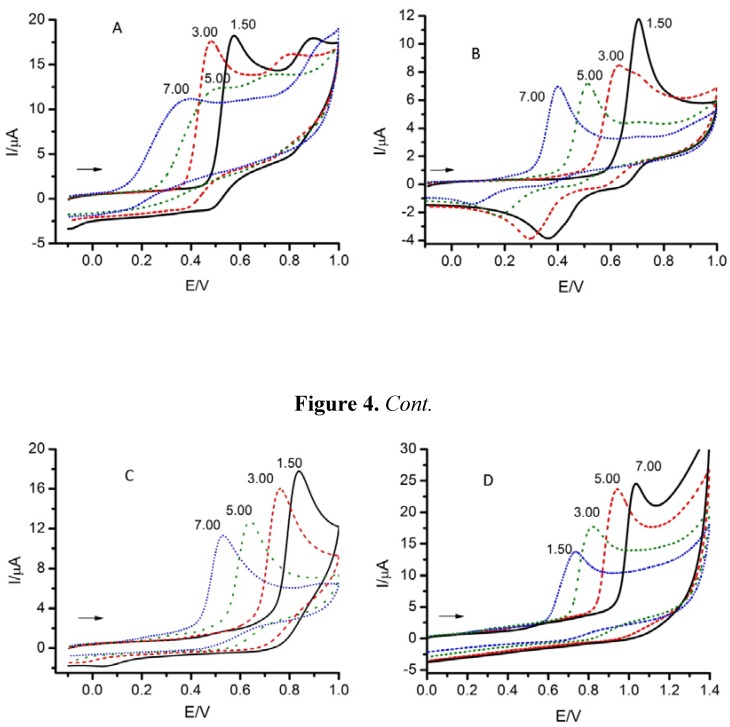
Cyclic voltammograms of A: gallic acid, B: eugenol, C: thymol, D: 3-hydroxycoumarin. Experimental conditions: v = 0.1 V·s^−1^, c = 5·10^−4^ M and different pH values. Arrows indicate the initial direction of the scan.

For a pure diffusion first-order process, the peak potential is not expected to vary with the concentration. So, the values of ∂E_p,a_/logc indicate that adsorption processes must complicate the electrochemical reactions, as it was concluded above. In most cases, as can be seen in Figure 1, the antioxidants studied here have groups that present acid-base characteristics such as –OH, –COOH or both. So, the effect of pH on the voltammetric response must be taken into account. For this reason, experiments were made at constant scan rate and reactant concentration, and varying the acidity of the medium, as is shown in Figure 4 for some selected substrates.

The optimal conditions to carry out the measurements for the determination of antioxidant activity can be obtained from the examination of these experimental results. So, it is important to perform the voltammetric measurements with the minimal charging current possible, because this implies that the extrapolation of the charging current to the potentials corresponding to the peak is more reliable. So, as it can be seen in Figure 2, this requirement implies the use of low scan rates, this being found for the rest of antioxidants studied. Nevertheless, it is also important to obtain the voltammetric curves with the minimal distortion, this implying not extremely low scan rates. On the other hand, the higher sensibility in intensity was intended and so, the concentration values (and also the scan rates) must be high.

Finally, in general as the pH was increased the oxidation potentials shifted towards less positive values (see Figure 4), this implying that the oxidation becomes easier. But high pH values imply the dissociation of weak acidic groups of the molecules and no physiological conditions. So, equilibrium between the above considerations let the authors to take as the optimal conditions to made the measurements those following: pH = 7, v = 0.1 V·s^−1^ and c = 5·10^−4^ M.

**Figure 5:**
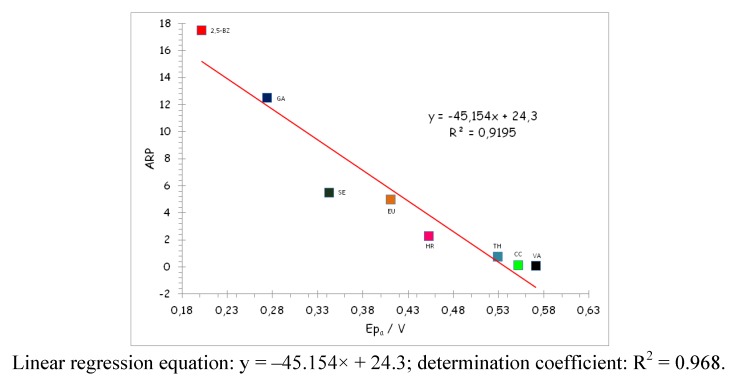
ARP-oxidation potentials correlation of the studied compounds at pH = 7.0, scan rate 0.1 V·s^−1^ and concentration 5 × 10^−4^ M.

The voltammograms corresponding to the different antioxidants studied in this paper under the above conditions show that the antioxidants exhibit well-defined oxidation voltammetric peaks and, in some cases, as EU or SE, a reduction peak, accompanying the oxidation one, is also found. The peak oxidation potential can be measured accurately for each compound, this being gathered in Table 1. The area of each voltammetric peak (which can be interpreted as a charge) is related with the concentration of antioxidant and with the specific oxidation mechanism, mainly with the number of electrons involved in the oxidation and thus it cannot be taken as a measurement of the antioxidant capacity of the compound in question. Figure 5 shows the correlation obtained between the ARP and the oxidation potential values of the most significant compounds of the study. As can be seen this correlation is good, having r = −0.984, a slope of −29.40 ± 2.68 and an intercept of 16.25 ± 1.17 (at a confidence level of 95%). From the above results it follows that the good correlation existing between oxidation potentials and ARP indicates that the voltammetric method can be used for the determination of antioxidant activity, in the same way as the DPPH^•^ assay. So, the values of the oxidation potentials can be interpreted in the same way as the ARP values obtained from DPPH^•^, that is, the quality of the information obtained is the same in both cases.

From a mechanistic point of view, the process involves the loss of electrons from the starting structure, related to the experimentally found potential value (E_p,a_). The addition of a single hydroxyl–group on a benzene (phenyl) ring has drastic consequences on the chemical properties of this otherwise quasi-inert aromatic system [28]. It is generally assumed that the initial stage in phenols, especially under conditions of pH close to 7.0, leads to the formation of a phenoxenium cation *via* phenoxy radical that subsequently evolves through other chemical processes such as loss of a proton, coupling reactions or nucleophilic substitutions [29]. Thus, phenolic species possessing –alkyl and/or –alkoxy substituents at *ortho* and *para* and/or carbonylic substituents at *meta-*, produce a drastic decrease in the potential values.

In our hands the phenolic compound with a lower potential (E_p,a_) value has been 2,5-BZ, that possess a *p*-aromatic phenoxyl group and a *m*-aldehyde; its isomer 2,4-BZ shows a higher E_p,a_ value since it has not the substituents in positions that favored its electrochemical activity: the aldehyde group in *para* and the alcohol in *meta*. In second place appeared GA, having neighboring phenoxyl groups which are prone to oxidation with formation of hydrogen peroxide, quinones, and semiquinones [30], and an acid group responsible for contributing to the stabilization of the specie resulting from the oxidative process. Next, phenolic compounds having *ortho* or *para* -alkyl or -alkoxy groups as SE, EU, HR (*p*-substituted) and TH, VA or CC (*o*-substituted).

The oxidation potential of a given compound candidate to have antioxidant activity has a limiting value of 0.56 ± 0.09 V (the value of the correlation at which the ARP is zero). This means that above this oxidation potential the investigated compound must not show antioxidant activity in the DPPH^•^ test. This is illustrated in Table 1: SA, CI and HC, for example, have ARP values lower than 10^–3^ (which, on the other hand, have low accuracy due to the uncertainty in the measurements), and CC is in the limit of considering the molecule as antioxidant. In the cases of CU and LI among others (Table 1), no antioxidant activity was found irrespective the DPPH^•^ assay or voltammetric measurements were used. This was also found in the literature in the investigation of free radical scavenging capacity of conducting polymers [31]. The high potentials of aniline and pyrrole monomers were related to less readily interaction with DPPH^•^, which has a formal potential of reduction 0.340 V *versus* Ag/AgCl at pH = 7 [32], that is, in the same conditions of the experiments here reported. This means that DPPH^•^ assay can assess radical scavenging for substances that are thermodynamically capable to react with agents with a redox potential below a given value, related to the reduction potential of DPPH^•^. Nevertheless, other less stable radicals of biological interest, as ROO^•^ and OH^•^, exhibit much higher formal potentials than DPPH^•^ [31] and can react with species having oxidation potentials that prevents the determination of the antioxidant activity by the DPPH^•^ assay.

An advantage of the electrochemical measurements over the DPPH^•^ assay is that oxidation potentials of the individual compounds can be determined with the same accuracy, irrespective the antioxidant character of such compounds, obviously provided that oxidation peaks are well defined. Thus, in the conditions of this work, the uncertainty in E_p_ is lower than ±3 mV. This is not true for the DPPH^•^ experiments because, in this determination, the lower the antioxidant activity, the higher the concentration needed and the longer the experiment, the variations in absorbance being subjected to more inaccuracy, this being the cause of the difference in ARP measurements reported by different laboratories for the same compound. At low antioxidant activities the results can be subject of a great uncertainty, as noted at the beginning of this section.

Other advantages of the electrochemical determination are that it is fast and cheap. Thus, a typical voltammetric experiment is made in less than 10 min. Moreover there is no necessity to use other reactants, lowering the cost. In addition, the low time of use of the instrumentation improves the optimization of research resources and, at the same time, the use of a small amount of organic solvents implies a minimum waste management.

Finally, conversely as occurs for the DPPH^•^ assay, the electrochemical measurements can be made at different pH values, as well as in different reaction media, this allowing the comparison between the antioxidant activity of candidate molecules under a variety of experimental situations such as physiological conditions, low pH environments as in the digestive system, *etc.*

## 3. Experimental

### 3.1. Reference Standards

Eugenol, sesamol, 3-hydroxycoumarin, cinnamaldehyde, 4-hexylresorcinol, carvacrol, 2,4-dihydroxybenzaldehyde, 2,5-dihydroxybenzaldehyde, 3,5-dimethoxybenzaldehyde, 4-methoxybenzyl alcohol, furfural, vanillin, cinnamic acid, ascorbic acid, 4-chloroanisole, geraniol, benzaldehyde, pulegone, menthol, 1,4-cineole, 2-furanacetic acid, menthone and salicylaldehyde were purchased from Aldrich; thymol, coumarin and limonene from Sigma and gallic acid from Sigma-Aldrich. 2,2-Diphenyl-1-picrylhydrazyl (DPPH^•^), free radical, 95% was purchased from Sigma-Aldrich. For the other compounds, Merck analytical grade reagents were used without further purification.

### 3.2. Cyclic Voltammetry

The working concentration of antioxidants was 5 × 10^−4^ M, with the exception of the experiments in which the influence of this variable was studied. Solutions of 0.1M in both acetic and phosphoric acids, for 1.5 < *pH* < 8, were used as supporting electrolyte. The aqueous solutions were prepared using ultrapure water type I (resistivity 18.2 MΩ.cm at 25 °C) obtained from an ultrapure water system Millipore Simplicity®. Antioxidants were dissolved in ethanol and the stock solutions were stored in darkness at 277 K to avoid decomposition. The concentration of ethanol in cell was 5%. Ionic strength was adjusted to 0.5 M with solid NaCl and the *pH* was adjusted with solid NaOH. Solutions were purged with purified nitrogen and the temperature was kept at 298 ± 0.1 K.

Measurements were made on a CHI650A electrochemical workstation from IJCambria. The working electrodes was a glassy carbon electrode from IJCambria (geometrical area = 38.5 mm^2^) which was cleaned by polishing with 0.3 µm alumina powder (Buehler Micropolish II). All potentials were measured against a Ag|AgCl|KCl_sat_ electrode (BAS MF-2052). A platinum counter electrode BAS MW-1034 was used.

### 3.3. Spectrophotometric Meausurements. DPPH^•^ Radical Scavenging Assay

The maximum wavelength of the UV-visible absorption band of the DPPH^•^ is 515 nm and the action of an antioxidant (AH) or a given radical (R^•^) causes the decrease of this band or its eventual disappearing through the following general reactions: 



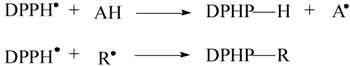
(1)


Caution was taken with this assay due, among other causes, to possible differences between structural and kinetic characteristics of the compounds, which could made necessary to investigate the interaction mechanism between each antioxidant and the DPPH^•^ radical. 

UV measurements were made on a Genesys 10 uv spectrophotometer from Thermo Electron Corporation with quartz cuvettes of path-length 1.0 cm.

Different concentrations of antioxidants were added to DPPH^•^ methanolic solution. The initial DPPH^•^ concentration was 6 × 10^−5^ M. The DPPH^•^ concentration in the reaction medium was calculated from a calibration curve with the equation:



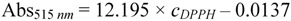
(2)


as determined by linear regression.

The amount of antioxidant required to decrease the initial concentration of DPPH^•^ to 50% (efficient concentration or EC_50_) measures the antioxidant activity. The reverse value, namely anti-radical power, ARP = 1/EC_50_, should be higher as the antioxidant is more efficient.

## 4. Conclusions

In conclusion, our study showed that antioxidant capacities of some active principles of spices and condiments belonging to the low molecular weight antioxidants (LMWA) family as deduced from CVs strongly correlate with those determined using a well-known spectrophotometric technique (DPPH^•^ assays). This good correlation between oxidation potentials and ARP indicates that the voltammetric assay can be used for the determination of antioxidant activity, in the same way as the DPPH^•^ assay. The method has been tested also for non-aromatic compounds with different characteristics such as acids, sugars or enzymes. The electrochemical measurements present several advantages over the DPPH^•^ assay, the most significant being that oxidation potentials of the individual compounds can be determined with the same accuracy, irrespective the antioxidant character of such compound and that it is faster and cheaper than the ARP determination. Moreover the electrochemical measurements can be carried out at different pH values, as well as in different reaction media, this allowing the comparison between the antioxidant activity of candidate molecules under a wide variety of experimental conditions. A limiting value of the oxidation potential of a given compound candidate to have antioxidant activity in the DPPH^•^ test was found, this being explained by the difference of the formal potentials of the antioxidant and the DPPH^•^ radical. 
